# Central Pulmonary Embolism Detected on a Chest X-Ray: A Case Report

**DOI:** 10.5334/jbsr.3052

**Published:** 2023-02-15

**Authors:** Thomas Saliba, Denis Tack

**Affiliations:** 1 ULB, BE; 2 Epicura Hospital, BE

**Keywords:** chest X-ray, pulmonary embolism, Fleischner’s sign, knuckle sign, Westermark sign

## Abstract

Patients presenting to the emergency room with respiratory symptoms often receive a chest X-ray as part of the initial workup to exclude common pathologies. A 60-year-old patient presented to the emergency department with dyspnoea. An initial chest X-ray revealed the Fleischner’s sign, the knuckle sign, and the Westermark sign, specific but not sensitive for central pulmonary embolism, prompting a follow-up angio CT to confirm the diagnosis. The patient was treated and discharged a few days later.

**Teaching point:**

Chest X-rays, done as part of an initial workup, can show signs of pathologies that are not yet clinically suspected, such as pulmonary embolism.

## Introduction

Pulmonary embolisms are frequently encountered in clinical practice, with an incidence of 50 to 200 per 100,000, and are a common cause for radiological exams [1]. Overall mortality due to acute pulmonary embolism ranges from 10–30%, causing 300,000 to 370,000 deaths each year in Europe alone, making it the third most common cardiovascular cause of death [1]. Chest radiographs are a frequently used as a first-line exam in patients suffering with respiratory symptoms, and due to pulmonary embolisms often presenting with non-specific signs and symptoms, these patients often fall into that category [2]. It is therefore important to possess the ability to notice findings suggestive of pulmonary embolism on a chest X-ray to raise the possibility of this diagnosis and then confirm it with further imaging.

## Case History

**Figure 1:**
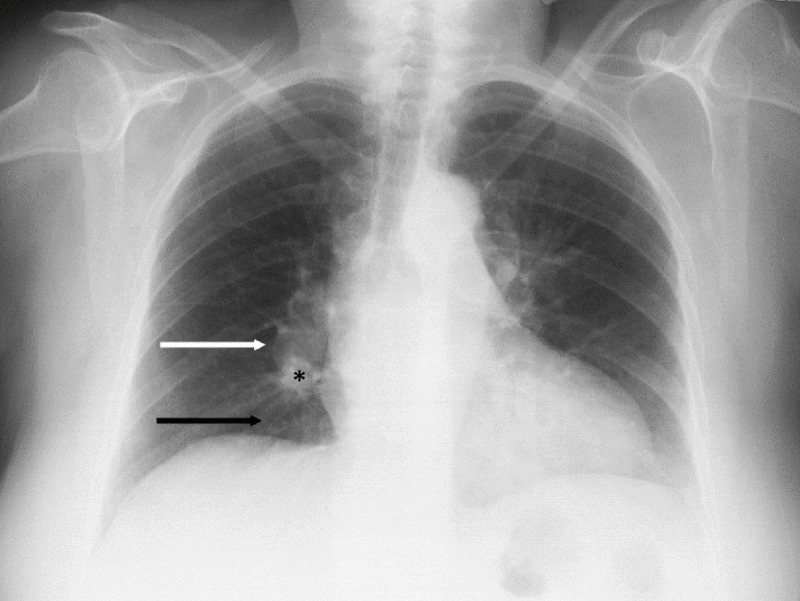
Chest X-ray demonstrating Fleischner’s sign (white arrow), Westermark’s sign (black arrow), and the knuckle sign (black star).

**Figure 2:**
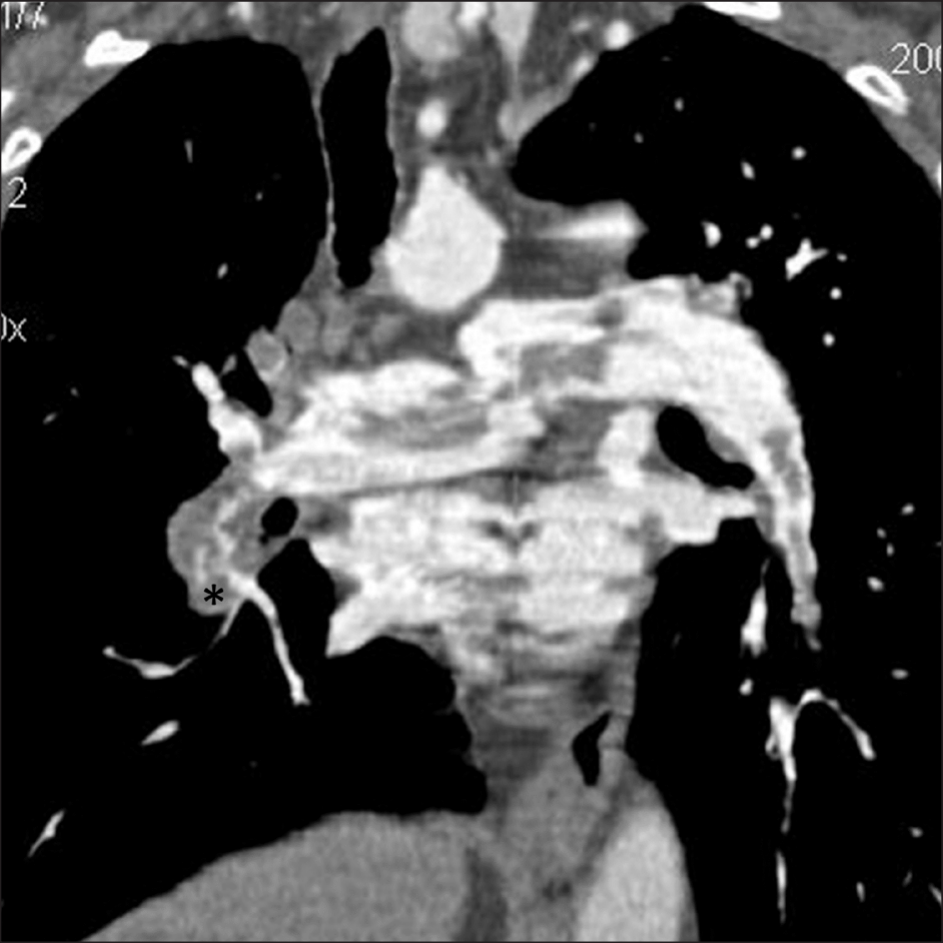
Coronal contrast-enhanced CT pulmonary angiography image demonstrating emboli filled and enlarged right intermediate pulmonary artery. The pulmonary artery abruptly tapers with due to the blood clot, whose distal end is marked (star).

We report the case of a 60-year-old woman presenting to the emergency department with dyspnoea. A chest X-ray was ordered as part of her initial workup, showing a concurrent prominent pulmonary artery, focal peripheral hyperlucency due to oligemia and an abrupt tapering of the pulmonary artery (Figure 1). The diagnosis of central pulmonary embolism without signs of infarction was suggested based on the X-ray, prompting the emergency room doctor to request a contrast-enhanced computed tomography (CT) exam, confirming the diagnosis (Figure 2). The patient was treated with anticoagulants and hospitalised for further treatment. The patient was treated for several days before being subsequently discharged with further anticoagulant treatment.

## Comments

The diagnosis of pulmonary embolism on chest X-rays is often a difficult task. In the case of this patient’s chest X-ray, three signs suggestive of embolism were visible. The first was a prominent pulmonary artery, Fleischner’s sign, which occurs due to pulmonary artery hypertension or due to a massive thrombus physically distending the artery [3,4]. The second visible sign was the sudden tapering of the pulmonary artery caused by reduced flow, the knuckle sign, often seen in tandem with Fleischner’s sign due to their linked origin [4,5]. The final sign was the focal peripheral hyperlucency caused by oligemia due to smaller clots, called Westermark’s sign [4,6]. These three signs, clearly visible on the chest X-ray (Figure 1), are not sensitive but are highly specific for central pulmonary embolism, without peripheral infiltration in this case [7]. Other possible evocative signs include pleural effusion, seen in around 35–55% of patients with acute pulmonary embolism, though the side of the effusion does not necessary indicate the side of the embolism [4]. Another possibility is Chang’s sign, described as a dilation of a previously normal descending pulmonary artery, usually within 24 hours of chest pain onset and maximally at two or three days later [8]. One may also see non-specific signs such as parenchymal consolidation or loss of volume, related to atelectasis or resulting from oedema or haemorrhage respectively [4]. In the case of infarction after embolism, this may present as a wedge-shaped peripheral consolidation, seen in 5–10% of patients, referred to as Hampton’s hump [4].

Once the suspicion of a pulmonary embolism is raised on a chest X-ray a follow-up angio CT exam can be performed to confirm the suspicion. The CT exam can be used to directly demonstrate the embolism, which will appear as a low-density clot within a pulmonary artery, standing in contrast to the pulmonary artery enhancement of the angio CT [1]. Furthermore, CT-exams can demonstrate signs of right heart failure, such as an enlarged pulmonary trunk (>29 mm), an increased right versus left ventricular diameter ratio (>1), contrast reflux into the vena cava or even bowing of the interventricular septum towards the left [1]. One can also see the tomodensitometric appearance of the aforementioned ‘Hampton’s hump’, which appears as a wedge-shaped peripheral opacity, with the possible addition of the ‘atoll’ sign, defined as a ground glass centre with a consolidated contour [1]. The differential diagnosis of the atoll sign includes fungal, bacterial, and mycobacterial infection as well as non-infectious disease, cancer, and changes resulting from treatment as well as pulmonary embolism [9]. The differential diagnosis of Hampton’s hump is essentially infection or cancer [10].

## Conclusion

The role of the radiologist is to not only to make the diagnosis that the clinician suspects but to make the diagnosis that which is not suspected. It is therefore important to be alert to signs of alternate diagnoses than those considered by the clinician. As pulmonary embolisms are a common and potentially life-threatening pathology it is essential to always be on the lookout for them. In this case we illustrate how a standard chest radiograph can tip-off the observant radiologist to signs of a pulmonary embolism, leading to a follow-up scan to confirm the diagnosis.
